# Trimethylamine N-oxide alleviates the severe aggregation and ER stress caused by G98R αA-crystallin

**Published:** 2009-12-19

**Authors:** Bo Gong, Li-Yun Zhang, Chi-Pui Pang, Dennis Shun-Chiu Lam, Gary Hin-Fai Yam

**Affiliations:** Department of Ophthalmology and Visual Sciences, The Chinese University of Hong Kong, Hong Kong, China

## Abstract

Purpose: Crystallins are major functional and structural proteins in mammalian lens. Their expression, distribution, and protein–protein interaction affect lens development and fiber cell differentiation. Mutated crystallins lead to structural and functional changes of lens structure and could lead to opacity formation and cataract development. The purpose of this study was to investigate the biological effects of the cataract-causing G98R mutation on the αA-crystallin (CRYAA) protein and to test the capability of chemical chaperone trimethylamine N-oxide (TMAO) to reverse such effects.

Methods: Myc/His-tagged, human, full-length, wild-type (WT) or G98R CRYAA was expressed in human lens epithelial B3 cells and treated or not treated with TMAO. Triton X-100 (Tx) solubility and cellular localization of CRYAA were examined by western blotting and confocal immunofluorescence, respectively. Ubiquitin proteasome-associated degradation was assayed by MG132 treatment. Endoplasmic reticulum (ER) stress, unfolded protein response, and apoptosis were analyzed by the expression of phosphorylated protein kinase-like ER-kinase, binding immunoglobulin protein (BiP), C/EBP homologous protein/growth arrest and DNA damage-inducible gene 153 (CHOP/GADD153), and caspase-3 and immunocytochemistry. Changes in heat shock and stress signaling were investigated.

Results: When transfected in lens epithelial B3 cells, unlike WT CRYAA located in the cytoplasm, the G98R CRYAA mutant formed aggregates inside the ER and the protein was predominantly Tx-insoluble. ER stress was induced by G98R CRYAA expression, and cells underwent apoptosis, as shown by a more frequent appearance of fragmented nuclei. Treatment with TMAO reduced Tx-insoluble mutant protein in time- and dose-dependent manners. Other chemical chaperones, 4-phenylbutyric acid, dimethysulfoxide, and glycerol, were much less effective than TMAO. ER-associated aggregates were reduced after TMAO treatment, and the protein was degraded through the ubiquitin–proteasome pathway. This alleviated ER stress and resulted in less apoptosis. Moreover, TMAO treatment induced a moderate upregulation of heat shock protein 70, indicating its effect on heat-shock response to modulate protein folding and assembly. No change was found for nontransfected cells after TMAO treatment.

Conclusion: The natural osmolyte and chemical chaperone TMAO reduced the aggregation of G98R CRYAA. This alleviated ER stress and rescued the affected cells from apoptosis. Our results showed that the chemical chaperone reduces mutant CRYAA aggregates in lens cells. We suggest a potential chemical-based strategy to reduce lens opacity formation.

## Introduction

Lens crystallins are a major family of water-soluble functional and structural proteins. Their expression, distribution, and protein–protein interaction are crucial for lens development and fiber cell differentiation [1]. Short-range ordering existing between the tightly packed native crystallin protein in lens fiber cells maintains a minimal solution turbidity and a high degree of light transmission, hence contributing to the transparency of lens [2]. Changes in structural and functional properties of crystallins could cause the breakdown of lens microstructure and result in a variation of the refractive index and light scattering [3]. Mutations in crystallin genes that cause the production of aggregated crystallins in the state of modified, damaged, or partially unfolded protein structures could lead to opacity formation and cataract development [4,5].

Insolubility of crystallin proteins and formation of large protein aggregates are the major factors that lead to lens opacity formation [6]. α-Crystallin is a predominant structural and functional protein, comprising up to 40% of lens proteins. It is composed of two subunits, αA- and αB-crystallin. Native αA-crystallin (CRYAA) is a member of the small heat-shock protein (hsp) family present in both ocular and nonocular tissues [7-9]. It exhibits chaperone-like activity to suppress the thermally induced aggregation of β- and γ-crystallins, the two major structural lens proteins [10-13], and to protect them from inactivation [14,15]. This capability underscores the importance of CRYAA in lens development and maintenance of transparency. Loss of chaperoning activity in mutant CRYAA results in the formation of aggregates encompassing its substrate proteins [16]. These intracellular protein aggregates adversely affect normal cell metabolism, lens fiber cell differentiation, and cell survival, leading to abnormal light scattering in lens structure and possibly cataract development [17-19]. Besides the W9X mutation causing almost complete truncation of protein [20], other known congenital cataract-causing CRYAA mutations (R12C, R21L, R49C, R54C, G98R, and R116C) are conserved in the modification of arginine residues. Hence, the encoded mutant proteins could have altered positive charges, conformational changes, and/or loss of chaperoning activity [21-27]. G98R CRYAA was reported to segregate with presenile onset cataract in an Indian family with disease onset at the age of 16 [25]. Purified G98R CRYAA protein was misfolded and aggregation prone due to the exposed hydrophobic peptide patches and lacked the corrective activity for DL-dithiothreitol (DTT)-induced aggregation of insulin [19,28]. In this study, we expressed mutant CRYAA proteins, including G98R, in human lens epithelial B3 cells to study their solubility, aggregation, localization in organelles, and apoptotic effects. We tested if the defective protein properties could be resolved by treatment with chemical chaperones.

## Methods

### Expression constructs and mutagenesis

A 548-bp EcoRI/XhoI fragment encompassing the full-length 519 bp open reading frame of *CRYAA* was ligated into the EcoRI/XhoI sites of a mammalian expression plasmid pcDNA6-His/myc (Invitrogen, Carlsbad, CA) to obtain the construct pHis/myc-CRYAA^WT^. This cloned fragment was used as a template to generate specific mutations of *CRYAA*, using QuikChange II Site-Directed Mutagenesis kit (Stratagene, La Jolla, CA) and oligonucleotides (Table 1). Wild-type (WT) and mutant constructs were verified by direct sequencing.

**Table 1:** Sense oligonucleotides for site-directed mutagenesis in *CRYAA*.

**Mutation**	**Oligonucleotides (sense, 5’ to 3’; bold underlined letters indicate specific base change)**	**Expression constructs**
R12C (c.34C>T)	ATCCAGCACCCCTGGTTCAAG**T**GCACCCTGGGGCCCTTCTAC	pHis/myc-CRYAA^R12C^
R21L (c.62C>T)	TGGGGCCCTTCTACCCCAGCC**T**GCTGTTCGACCAGTTTTTCG	pHis/myc-CRYAA^R21L^
R49C (c.145C>T)	TCCACCATCAGCCCCTACTAC**T**GCCAGTCCCTCTTCCGCACCG	pHis/myc-CRYAA^R49C^
G98R (c.292G>A)	GTGGAGATCCAC**A**GAAAGCACAACGAG	pHis/myc-CRYAA^G98R^
R116C (c.346C>T)	ACATTTCCCGTGAGTTCCAC**T**GCCGCTACCGCCTGCCGTCC	pHis/myc-CRYAA^R116C^

### Cell culture and transfection

Human lens epithelial B3 cells (American Tissue Culture Collection, Manassas, VA) were cultured in Eagle’s Minimal Essential medium (Invitrogen) supplemented with 10% fetal bovine serum (Invitrogen) and antibiotics (100 units/ml penicillin G and 100 μg/ml streptomycin sulfate, Invitrogen). Cells (10^5^ cells/cm^2^) were transfected with CRYAA constructs by using FuGene HD transfection reagent (Roche, Basel, Switzerland) at an optimized ratio of 3 μl FuGene per μg DNA in Opti-MEM^®^ I (Invitrogen) supplemented with GlutaMAX^TM^-I (Invitrogen). Chemical chaperone treatments were started at 24 h after transfection.

### Treatment by trimethylamine N-oxide and other chemical chaperone molecules

Trimethylamine N-oxide (TMAO) (25–300 mM; Sigma, St Louis, MO), 4-phenylbutyric acid (4-PBA; 0.2–2 mM; tributyrate; Triple Crown America Inc, Perkasie, PA), glycerol (1–5%; Sigma), or dimethylsulfoxide (DMSO; 0.5–1%; Sigma) was added to the transfected cells. Fresh culture medium containing drugs was replenished every 2 days.

### Protein analysis

Cells were lysed at a concentration of 2.5×10^6^ cells/ml radioimmunoprecipitation (RIPA) buffer containing 50 mM Tris-HCl (Sigma), 150 mM sodium chloride, 1% Nonidet P-40 (Sigma), 0.25% sodium deoxycholate (Sigma), protease inhibitor cocktail (Roche), and 1 mM phenylmethyl sulfonylfluoride (PMSF; Sigma) for 30 min on ice. After centrifugation, the supernatant was collected and denatured in sample buffer with a final concentration of 2% sodium dodecyl sulfate (SDS; BioRad, Hercules, CA) and 50 mM DTT (Sigma). The cell pellet was washed with ice-cold PBS and denatured in SDS sample buffer containing 9 M urea (BioRad).

For Triton X-100 (Tx) solubility analysis, cells were washed twice with ice-cold PBS and added to lysis buffer, which contained 100 mM Tris-HCl (pH 7.4), 3 mM ethylene glycol tetraacetic acid (EGTA; Sigma), 5 mM MgCl_2_, 0.5% Triton X-100 (Tx; Sigma), protease inhibitor cocktail, and 1 mM PMSF at 5×10^6^ cells/ml, for 2 min on ice. After centrifugation, the supernatant containing Tx-soluble protein was denatured in SDS buffer. The pellet containing Tx-insoluble protein was washed twice with ice-cold PBS, sonicated, and denatured in urea-SDS buffer [29]. The samples were analyzed with 10% SDS-polyacrylamide gel electrophoresis and western blotted using horseradish peroxidase (HRP)-conjugated antibodies against myc (BD Biosciences, San Jose, CA) recognizing CRYAA, glyceraldehyde-3-phosphate dehydrogenase (GAPDH; Sigma), or β-actin (Sigma), or monoclonal antibodies against binding immunoglobulin protein (BiP; BD BioSciences), C/EBP homologous protein/growth arrest and DNA damage-inducible gene 153 (CHOP/GADD153; Santa Cruz Biotech, Santa Cruz, CA), caspase-3 (Santa Cruz Biotech), or phosphorylated ER kinase (PERK; Santa Cruz Biotech), followed by appropriate HRP-conjugated immunoglobulin (Ig) secondary antibodies. Signals were detected by enhanced chemiluminescence (Amersham, Bucks, UK).

### Immunofluorescence

Cells were fixed with 2% neutral buffered paraformaldehyde (Sigma) in 0.1 M PBS, permeabilized and detected with mouse monoclonal anti-myc (recognizing CRYAA; Sigma) or anti-protein disulfide isomerase (PDI) antibody (BD BioSciences) followed by appropriate fluorescence-conjugated IgG secondary antibody (Invitrogen) and 4'-6-diamidino-2-phenylindole (DAPI; Sigma) staining. For semiquantitative evaluation of cytoplasmic CRYAA aggregates, >200 cells per sample were analyzed. We graded CRYAA aggregation from 0 to 3+: 0, no aggregate; 1+, few dot-like aggregates; 2+, more dot-like aggregates, and 3+, extensive large aggregates. The staining index, indicative of phenotype severity, was calculated using the formula: 4(3+)% + 2(2+)% + 1(1+)% [30].

### Transcription analysis

Total RNA was obtained by an RNA purification kit (RNeasy kit, Qiagen, Valencia, CA) and an on-column DNase digestion kit (RNase-free DNase kit, Qiagen). cDNA from 1 μg RNA, 10 ng/ml random hexanucleotide primer (Invitrogen), and reverse transcriptase (SuperScript III, Invitrogen) was amplified for CRYAA, hsp70, and hsp90 as well as other stress markers (Table 2).

**Table 2:** Expression primer sequences.

**Gene**	**GeneBank accession number**	**Specific primer sequences (5’ to 3’)**	**Product size (bp)**
αA-crystallin (*CRYAA*)	U05569	F: CGGGACAAGTTCGTCATCTT	203
		R: GCAGACAGGGAGCAAGAGAG	
Heat-shock protein 70 (*Hsp70*)	NM05345	1F: AAGTACAAAGCGGAGGACG	249
		1R: GATGGGGTTACACACCTGC	
		2F: TGCTGATCCAGGTGTACGAG	185
		2R: CGTTGGTGATGGTGATCTTG	
Heat-shock protein 90α (*Hsp90α*))	NM005348.3	F: ACCCAGACCCAAGACCAACCG	141
		R: ATTTGAAATGAGCTCTCTCAG	
Superoxide dismutase (*SOD*)	NM000454.4	F: AGGGCATCATCAATTTCGAGC	430
		R: CAAGGGAATGTTTATTGGGCG	
αB-crystallin (*CRYAB*)	NM001885.1	F: TCACCTAGCCACCATGGACATCGCCA	541
		R: CAAAAGCTTATTACTATTTCTTGGGGG	
Metallothionein 1M (*MT*)	NM176870.2	F: GCAAAGAGTGCAAATGCACCTC	125
		R: TCAGGCACAGCAGCTGCACT	
Interleukin-6 (*IL-6*)	NM000600.2	F: CTGGTCTTTTGGAGTTTGAGGTATACC	295
		R: CCATGCTACATTTGCCGAAGA	
Glyceraldehyde 3-phosphate dehydrogenase (*GAPDH*)	BC014085	F: GAAGGTGAAGGTCGGAGT	225
		R: GAAGATGGTGATGGGATTTC	

### Terminal apoptosis assay

Fixed cells were stained for myc and red X-conjugated secondary antibody (Jackson ImmunoRes Lab, West Gloves, PA), and nuclei were counterstained with DAPI. Samples were examined by fluorescence microscopy (DMRB microscope; Leica, Wetzlar, Germany) equipped with a color imaging system (Spot RT, Diagnostic Instruments, Sterling Heights, MI). Terminal apoptosis rate was represented as percentage of cells with fragmented nuclei. For each experiment (n=3), ten random images (40× objective) were analyzed.

## Results

### Expression of cataract-causing mutant CRYAA variants in B3 cells

**Figure 1:**
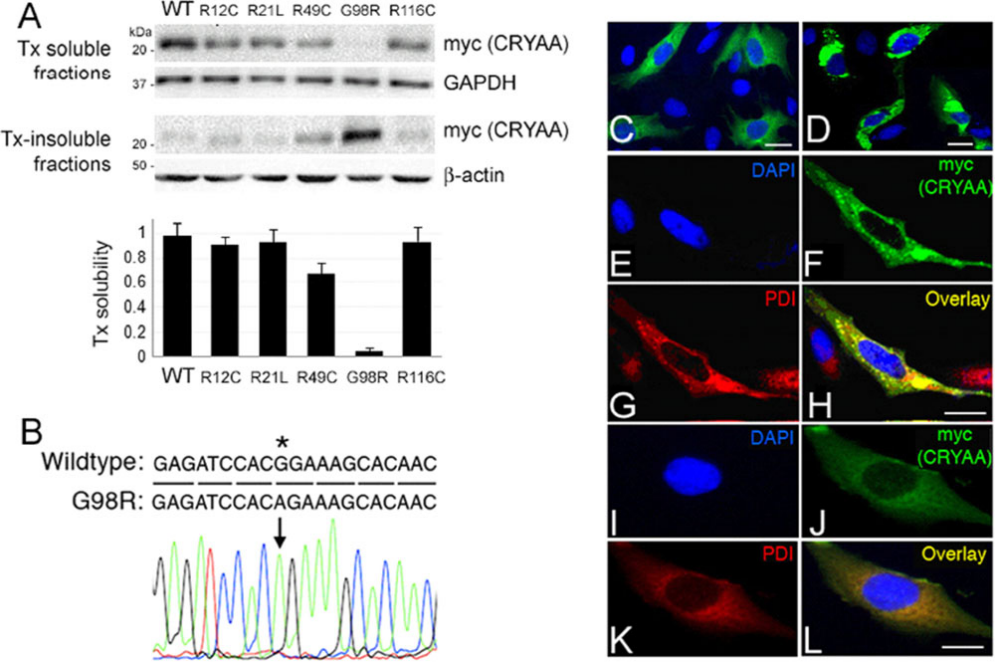
G98R αA-crystallin was TritonX-100-insoluble and formed aggregates inside cells. **A**: Triton X-100 (Tx) solubility assay of wild-type (WT) and cataract-causing mutant CRYAA in B3 cells by western blotting of myc (detecting CRYAA), housekeeping GAPDH, and β-actin. The band densitometry analysis showed the drastic reduction of Tx solubility of G98R CRYAA when compared to WT or other mutants. **B**: Direct sequencing of pHis/myc-CRYAA^G98R^ to indicate the base change at c.292G>A. **C**–**L**: Confocal double immunofluorescence of WT and G98R CRYAA in B3 cells. **C** and **D**: A lower magnification to show the expression of WT (**C**) and G98R CRYAA (**D**) in cells. **E**–**H**: G98R CRYAA (myc staining in **F**) formed intracellular aggregates and was intensely co-distributed with PDI (**G**) in the overlay image (**H**). **I**–**L**: WT CRYAA (**J**, myc staining) was diffusely distributed in cytoplasm and had only mild co-distribution with PDI (**K**) in overlay image (**L**). Nuclei were stained with DAPI (blue, **E** and **I**). Scale bars: 10 μm (**C**–**L**). PDI: protein disulphide isomerase; DAPI: 4'-6-diamidino-2-phenylindole.

A repertoire of congenital cataract-causing CRYAA mutants (R12C, R21L, R49C, G98R, and R116C) and WT CRYAA were cloned into the pcDNA6-His/myc expression vector and verified by sequencing. Each construct was then transfected to human lens epithelial B3 cells. At 48 h, the cells were collected in 0.5% Tx lysis buffer and fractionated to Tx-soluble and Tx-insoluble proteins. By western blotting for myc, representing CRYAA, most His/myc-tagged CRYAA mutants were Tx-soluble, whereas His/myc-G98R CRYAA was predominantly Tx-insoluble (Figure 1A). By band densitometry analysis, the average Tx solubilities were 98% for WT CRYAA, 95% for R12C, 96% for R21C, 68% for R49C, 96% for R116C, and 1.6% for G98R CRYAA in B3 cells. Hence, the solubility of G98R CRYAA was substantially reduced when compared to WT and other known mutants. This insolubility was consistently observed upon transfection with different ratios of pHis/myc-CRYAA^G98R^ DNA to FuGene reagent. The transfection efficiency was similar among WT and all studied mutants, as verified by immunofluorescence of myc, representing CRYAA. The efficiency was maintained at about 50% at day 2 for sample collection.

### Localization of G98R CRYAA in B3 cells

The immunofluorescence study showed that His/myc-G98R CRYAA formed large-sized cytoplasmic aggregates (Figure 1D), whereas WT CRYAA was located diffusely in the cytoplasm (Figure 1C). To localize the His/myc-G98R CYRAA, we performed confocal double immunofluorescence. The aggregated G98R CYRAA was intensively co-localized with PDI, an ER resident protein (Figure 1E-H). In contrast, WT CRYAA exhibited mild co-distribution with PDI. This demonstrated for the first time that mutant CRYAA formed insoluble aggregates in the ER. Hence, we suggested that the misfolded mutant G98R CRYAA-induced presenile cataract could be an ER storage disease.

### G98R CRYAA aggregated in cytoplasm but reduced by trimethylamine N-oxide

**Figure 2:**
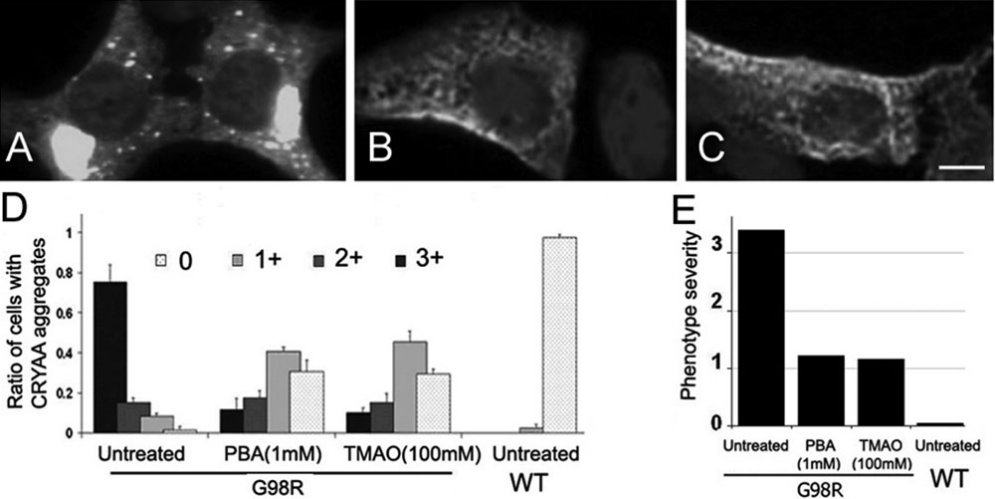
Chemical chaperones reduced the cellular aggregation of G98R αA-crystallin. **A**-**C**: Distribution of G98R αA-crystallin (CRYAA) in transfected B3 cells. Untreated B3 cells showed aggregated G98R CRYAA (**A**), which was reduced after 4-PBA (1 mM; **B**) and TMAO (100 mM; **C**) treatments. **D**: Semiquantitative scoring of intracellular CRYAA aggregates in cells. **E**: Staining index representing phenotype severity showed dramatic reduction after 4-PBA or TMAO treatment. 4-PBA: 4-phenylbutyric acid; TMAO: trimethylamine N-oxide

We hypothesized that the misfolding of G98R CRYAA with the exposed hydrophobic peptide patches could be rectified by treatment with TMAO, which is an osmolyte with a hydrophobic nature and acts as a chemical chaperone to stabilize proteins with a tightly packed conformation. To authenticate this action, we also tested other small molecule chemicals, 4-PBA, glycerol, and DMSO, by virtue of the chaperoning activity reported previously [31-37]. After treatment for 2 days separately with these chemicals, we examined the distribution of transfected CRYAA. We found that both TMAO and 4-PBA treatments resulted in fewer cells exhibiting large-sized myc-positive aggregates (Figure 2B,C compared to untreated control in Figure 2A). The majority of these mutant cells had cytoplasmic punctate staining. A scoring analysis showed 10% of the TMAO-treated mutant cells and 12% of the 4-PBA-treated mutant cells with a 3+ level of aggregation (Figure 2D). This was highly contrasted to approximately 75% of mutant cells before treatment (graded as 3+). The staining index, representing the phenotype severity, was also dramatically reduced after TMAO or 4-PBA treatment, approaching that of WT-expressing cells (Figure 2E). Treatment with 0.5 or 1% DMSO was ineffective in reducing His/myc-G98R CRYAA aggregates. Also, toxicity was observed in cells treated with 1% or 5% of glycerol.

### Trimethylamine N-oxide reduced Triton X-100-insoluble G98R CRYAA

**Figure 3:**
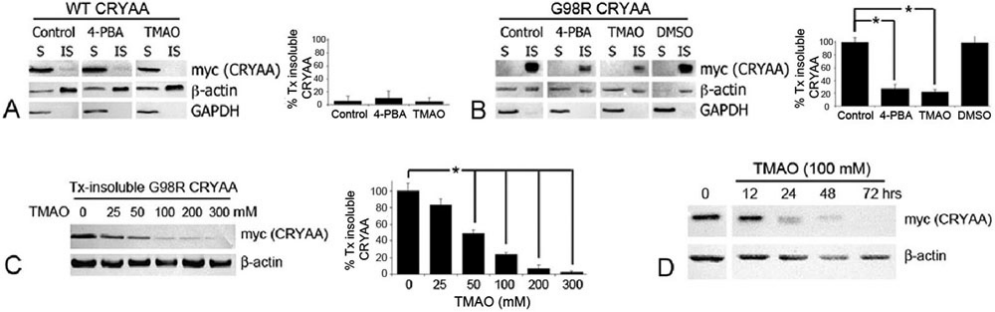
Trimethylamine N-oxide reduced the detergent insolubility of C98R αA-crystallin (CRYAA). **A**: Western blot analysis of myc (CRYAA) showed that the WT was mainly Tx-soluble and not affected by 4-PBA or TMAO treatment. Untreated G98R CRYAA was predominantly Tx insoluble, which was significantly reduced after 4-PBA (1 mM) or TMAO (100 mM) treatment for 2 days. The asterisk indicates a p<0.05 by independent Student * t *test. DMSO (1%) did not affect mutant insolubility. **B**: TMAO reduced Tx-insoluble G98R CRYAA dose dependantly. Treatment with TMAO (50 mM or higher) for 2 days significantly decreased Tx-insoluble G98R CRYAA. The asterisk indicates a p<0.05 by independent Student *t *test. **C**: TMAO (100 mM) reduced Tx-insoluble G98R CRYAA in a time-dependant manner. WT: wild-type; Tx: Triton X-100; 4-PBA: 4-phenylbutyric acid; TMAO: trimethylamine N-oxide; DMSO: dimethylsulfoxide.

Treatment with 100 mM TMAO or 1 mM 4-PBA for 2 days reduced Tx-insoluble His/myc-G98R CRYAA, whereas the expression of His/myc-WT CRYAA was unaffected (Figure 3A). The percentage of Tx-insoluble mutant protein after TMAO or 4-PBA treatment was similarly decreased to almost one-fourth of that in untreated cells (22% for TMAO treatment and 28% for 4-PBA; p<0.05, independent Student *t *test; Figure 3B). When examined at 5 days, 4-PBA-treated cells had notable morphological changes, whereas TMAO showed a slender fibroblast-like morphology at more than 5 days, which could be maintained for about 10 days. Due to the undesirable morphological changes, 4-PBA was not selected in further experiments. Treatment with 0.5% or 1% DMSO was again ineffective in reducing the insoluble His/myc-G98R CRYAA (Figure 3B), and 1% or 5% glycerol killed cells. Hence, DMSO and glycerol were not used in subsequent experiments.

A 2-day treatment with TMAO at concentrations from 25 to 300 mM led to a dose-dependent decrease of Tx-insoluble His/myc-G98R CRYAA (Figure 3C). TMAO at 25 mM reduced Tx-insoluble G98R CRYAA to 83% (compared to 98% for untreated mutant protein). Tx-insoluble G98R CRYAA was further reduced to 48% at 50 mM and to 24% at 100 mM treatment. The reduction was statistically significant (p<0.05, independent Student *t *test). Cell death became prevalent upon treatment with TMAO at concentrations higher than 200 mM. We further observed a time-dependent decrease of Tx-insoluble His/myc-G98R CRYAA when cells were treated with 100 mM TMAO (Figure 3D). At 12 h, the level decreased to 67% (compared to 98% for untreated mutant protein). It was further reduced to 28% at 24 h, 21% at 48 h, and 3% at 72 h, and the reduction was statistically significant (p<0.05, independent Student* t *test). It is notable that while the insoluble G98R CRYAA decreased by TMAO treatment, the level of soluble G98R did not increase. Hence, we postulated that TMAO could reduce the misfolded polypeptides of CRYAA via degradation rather than by refolding them.

### Trimethylamine N-oxide induced G98R CRYAA degradation by ubiquitin-proteasome pathway (UPP)

**Figure 4:**
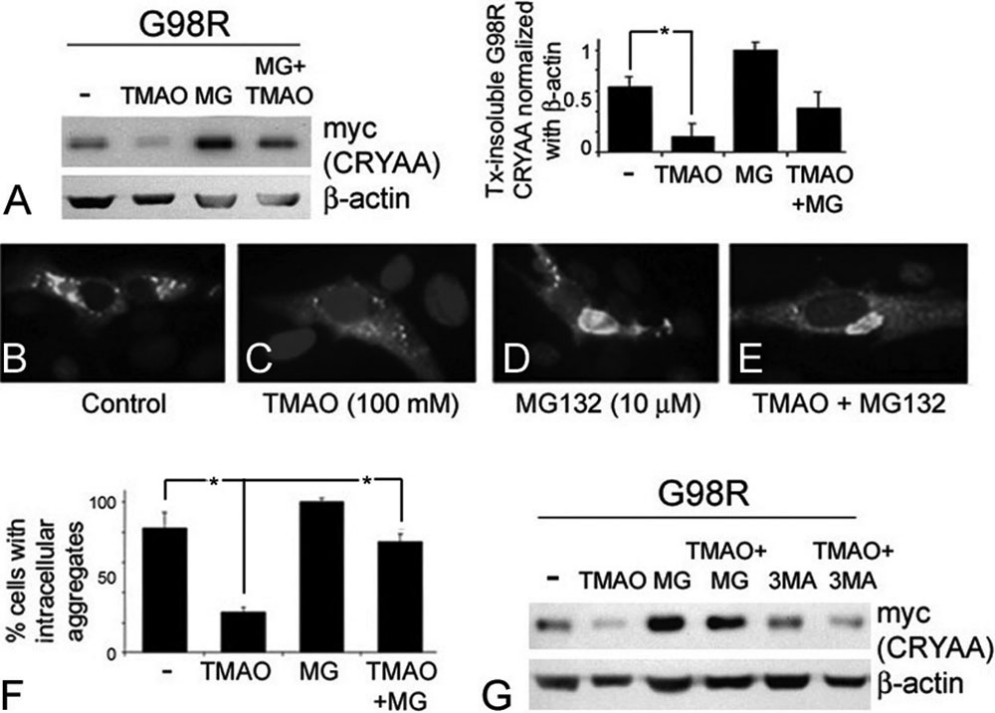
Trimethylamine N-oxide treatment degraded reduced G98R αA-crystallin (CRYAA) via the ubiquitin-proteasome pathway. **A**: Reduced level of Tx-insoluble G98R CRYAA after TMAO (100 mM, 2 days) was reversed by MG132 (10 μM, 8 h). MG132 alone further increased Tx-insoluble mutant protein. **B**–**E**: Cytoplasmic G98R CRYAA aggregates were reduced after TMAO treatment (**C**). Intense aggregation was observed in cells treated with TMAO and MG132 (**E**), MG132 only (**D**), and untreated cells (**B**). **F**: Percentages of cells with CRYAA aggregates after treatments. **G**: Null changes of Tx-insoluble G98R CRYAA after treatment with 3-MA, unlike that of MG132 treatment. The asterisk indicates a p<0.05 by independent Student *t *test. Tx: Triton X-100; TMAO: trimethylamine N-oxide; UPR: unfolded protein response; PERK: phosphorylated protein kinase-like ER-kinase; BiP: binding immunoglobulin protein; CHOP/GADD153: C/EBP homologous protein/growth arrest and DNA damage-inducible gene 153; WT: wild-type.

We next investigated how TMAO reduces Tx-insoluble His/myc-G98R CRYAA and resolves protein aggregates in the ER. In the presence of 10 μM MG132, a reversible inhibitor of UPP, in the transfected cell culture for 8 h, a substantial increase of Tx-insoluble His/myc-G98R CRYAA protein was observed (Figure 4A), and the reduced level of Tx-insoluble G98R CRYAA after TMAO (100 mM, 2 days) was reversed by MG132 (10 μM, 8 h). This indicated that G98R CRYAA protein could be degraded via UPP. Blocking of UPP resulted in more insoluble protein and augmented the mutant CRYAA aggregation in cells, as demonstrated by immunofluorescence (Figure 4D, compared to non-MG132-treated G98R cells in Figure 4B). After treatment with 100 mM TMAO for 2 days, a low level of Tx-insoluble mutant was again observed (Figure 4A). The levels were similar as in previous experiments. Further incubation of these TMAO-treated cells with 10 μM MG132 for 8 h increased Tx-insoluble CRYAA protein. This was not significantly different from the untreated mutant cells. Immunofluorescence showed that His/myc-G98R CRYAA aggregates reappeared inside cells (Figure 4E, compared to TMAO-treated cells in Figure 4C). We semiquantified the cells with visible intracellular G98R CRYAA aggregates. Nearly all MG132-treated mutant cells showed His/myc-CRYAA aggregates (Figure 4F). The simultaneous treatment with TMAO and MG132 resulted in 73% of cells with aggregates, which was similar to the untreated mutant cells (81%). Only TMAO treatment significantly reduced the cells with CRYAA aggregates to 36% (p<0.05 when compared to untreated cells or cells with both TMAO and MG132 treatments). On the other hand, treatment with 10 μM 3-methyladenine (3-MA, an inhibitor of autophagy) did not show any change of His/myc-G98R CRYAA solubility and subcellular aggregation (Figure 4G), indicating that CRYAA was not a substrate of autophagy. In addition, the solubility and subcellular aggregation of WT CRYAA was not affected by treatment (data not shown).

### Endoplasmic reticulum stress and apoptosis caused by aggregated G98R CRYAA were alleviated by trimethylamine N-oxide

**Figure 5:**
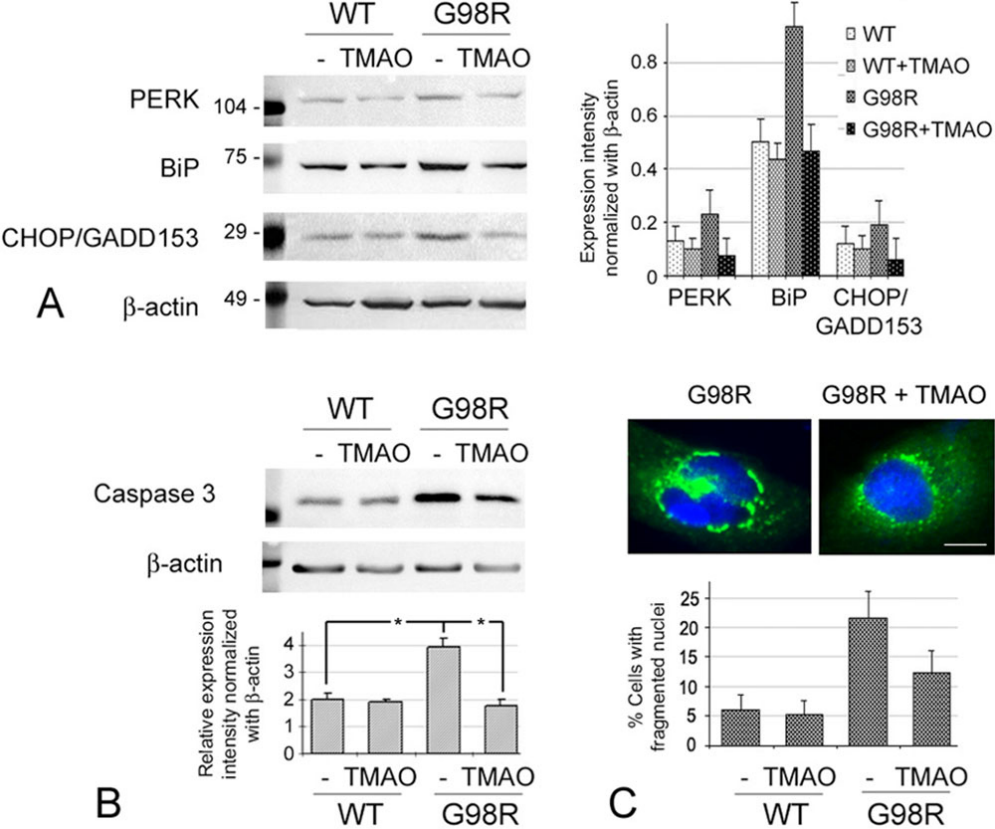
Trimethylamine N-oxide treatment alleviated the endoplasmic reticulum (ER) stress and apoptosis of cells expressing G98R αA-crystallin (CRYAA). **A**: TMAO (100 mM, 2 days) reduced expression of ER stress and UPR markers (PERK, BiP, and CHOP/GADD153) that were upregulated in G98R CRYAA cells. No specific change was observed for WT cells. **B**: Reduced caspase-3 expression in G98R CRYAA cells treated with TMAO. The asterisk indicates a p<0.05 by independent Student *t *test. **C**: Fewer G98R CRYAA cells exhibited fragmented nuclei after TMAO treatment.

We examined if TMAO affected the expression of ER stress response proteins, including PERK and BiP, in transfected B3 cells. These proteins were upregulated upon His/myc-G98R CRYAA expression when compared to WT CRYAA (Figure 5A). The expressions of PERK and Bip in mutant-expressing cells were increased about twofold compared to the WT-expressing cells (PERK from 13% to 22%; BiP from 51% to 92%). B3 cells incubated with a transfection reagent had no observable changes. There was, therefore, induction of ER stress due to mutant protein expression. With TMAO (100 mM) treatment for 2 days, the ER stress marker expression was substantially reduced to a level about one-half of the untreated cells (p<0.05; Figure 5A). The expression of PERK decreased from 22% to 7% and that of Bip decreased from 92% to 47%. WT-expressing cells were not affected by TMAO treatment, indicating that this chemical chaperone acted on proteins that were not natively folded.

We further explored the consequence on apoptotic cell death. Western blot analysis showed that CHOP/GADD153 and caspase-3 were upregulated in His/myc-G98R CRYAA-expressing B3 cells nearly twofold compared to WT-expressing cells (CHOP from 13% to 19%, caspase-3 from 20% to 29%; p<0.05; Figure 5A,B). This was accompanied by an increased percentage of cells undergoing apoptosis and showing fragmented nuclei revealed by double immunofluorescence of myc (representing CRYAA) and nuclear dye DAPI (G98R CRYAA cells 22% versus WT CRYAA cells 6%; Figure 5C). Treatment with TMAO (100 mM) for 2 days resulted in reduced expression of both CHOP/GADD153 and caspase-3 (p<0.05; Figure 5A,B). The expression of CHOP was decreased from 19% to 6% and that of caspase-3 from 2% to 18%. Consistent results were obtained in experiments done in triplicate. The suppression of apoptotic proteins was supported by a lower percentage of cells with fragmented nuclei in TMAO-treated G98R CRYAA cells (13% versus 22% in untreated G98R CRYAA cells; Figure 5C). TMAO treatment of WT cells did not induce changes in apoptotic protein expression or in amounts of apoptotic cells (Figure 5B,C).

### Effect of trimethylamine N-oxide treatment on cell stress signaling

**Figure 6:**
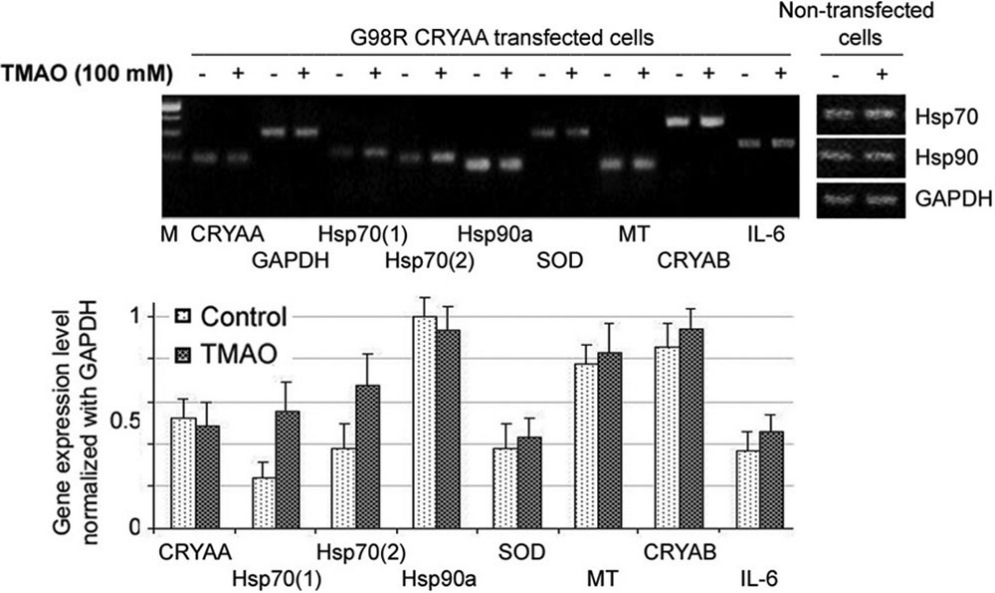
Heat-shock response and cell stress signaling. Semiquantitative reverse transcription-polymerase chain reaction analysis showed specific upregulation of *Hsp70* in G98R CRYAA cells treated with TMAO, whereas other stress-inducible genes remained unchanged. No changes were found for TMAO treatment on nontransfected cells. Hsp70: heat-shock protein 70; CRYAA: αA crystallin; TMAO: trimethylamine N-oxide

We sought to examine if TMAO treatment (100 mM, 48 h) affected pathways of stress signaling on G98R CRYAA cells. We studied gene expressions involved in heat-shock responses, including *Hsp70* and *90α*, and cell stress responses. By semiquantitative PCR we confirmed an upregulation of *Hsp70* by using two different pairs of specific primers, Hsp70 (1) and Hsp70 (2) (Figure 6). No change was observed for Hsp90α expression. In nontransfected cells, *Hsp70* and *Hsp90* expression was not altered by TMAO treatment (Figure 6). On the other hand, gene expression of cell stress signaling (superoxide dismutase *SOD* and αB-crystallin *CRYAB* for oxidative stress, metallothionein *MT* for heavy metal-induced stress, and interleukin-6 *IL6* for inflammatory-associated stress) was unaffected by TMAO.

## Discussion

In this study, we expressed the presenile cataract-causing G98R CRYAA in lens epithelial B3 cells by transfection. The mutant protein was found to be insoluble upon Tx extraction and prone to be retained and form aggregates in the ER of cells. This induced ER stress, and cells underwent apoptosis. This is the first time it has been shown that the misfolded G98R CRYAA-induced presenile cataract could be an ER storage disease. This cellular defect was corrected by treatment with a chemical chaperone, the natural osmolyte TMAO. Upon treatment, the Tx insolubility was reduced dose and time dependently, and the cells had less mutant protein aggregates, probably degraded via the ubiquitin proteasome pathway. TMAO treatment alleviated the ER stress and apoptosis. We also showed that TMAO treatment modulated hsp70 expression, which could be an underlying mechanism of how this chemical chaperone affected the protein folding environment in cells.

Unlike WT CRYAA, cataract-causing G98R CRYAA protein was exclusively partitioned to the Tx-insoluble fraction and formed inclusion bodies when expressed in bacterial cells [19]. We extended this observation to human lens epithelial B3 cells and discovered that Tx-insoluble G98R CRYAA mutant protein formed large ER-associated aggregates. The mutation from glycine to arginine induces a gain of positive charge, and the encoded protein is not optimally folded. The Bis-ANS binding assay has illustrated that the mutant protein has exposed hydrophobic sites that cause it to become aggregation prone with the formation of large mixed oligomers with WT CRYAA [19]. In our experiment we further showed that aggregation of His/myc-G98R CRYAA induced ER stress, as demonstrated by PERK and BiP upregulation. The mutant protein was modestly degradable by UPP. Application of the proteasome inhibitor MG132 resulted in an increased level of Tx-insoluble mutant protein. The prolonged ER overloading ultimately caused the cells to undergo apoptosis through a caspase-3-dependent pathway, in association with increased CHOP/GADD153 expression. Our novel observation underlines the aggregation of mutant CRYAA and subsequent cell death as a major pathogenetic factor for the development of lens opacity in cataract. On the other hand, other mutants, except R49C localized in both nuclear and cytoplasmic compartments [23], were mainly restricted in the cytoplasm without aggregate formation [38]. This delineates the severity of the highly aggregation-prone G98R mutant in cataract formation, and ways to reduce aggregation could alleviate the disease phenotype.

In recent years chemical chaperoning on misfolded proteins has been shown to prevent or correct protein’s non-native conformation, alleviating mistrafficking and the associated cellular defects, which resulted in enhanced cell survival [39-42]. Although the exact mechanisms are still undefined, chemical chaperones likely shift the folding equilibrium of substrate proteins to a more native state, reduce nonproductive aggregation, or enhance the resident chaperoning environment, allowing the misfolded proteins to break away from ER retention and facilitate the transport across intracellular compartments. This has been shown in correcting a variety of misfolded proteins associated with pathologic conditions and renders the chaperone-assisted protein rescue an appealing strategy to treat protein-folding diseases [34,35,43]. Removal of aberrant accumulation of misfolded proteins reduced cellular toxicity and enhanced cell survival [34,44-46].

In this study, we applied TMAO and other chemical chaperones that have been reported previously for eye-associated proteins to WT or G98R-CRYAA-expressing cells. Despite glycerol and DMSO causing significant cell death, TMAO and 4-PBA at the usage levels were nontoxic, which agreed with previous observations [47]. However, 4-PBA was not optimal due to the fibroblastic morphology of treated cells after long-term incubation. TMAO at optimal concentrations of 50–100 mM reduced Tx-insoluble CRYAA and resolved His/myc-G98R CRYAA aggregates in cells. These dosages were similar to our previous report on correcting the mutant myocilin [46]. The drug effect was mediated through UPP-associated degradation and was reversed by MG132 inhibition. It has been known that proper UPP function is essential for lens fiber cell differentiation [48], in addition to its general regulation in protein quality control, removal of obsolete proteins, cell cycle progression, DNA repair, immune responses, and so on [49]. Defective UPP leads to abnormal protein degradation, affecting protein biosynthesis and processing, and more severely, causes atypical protein accumulation and stress induction. This has been implicated in different human diseases, such as Parkinson’s disease, Alzheimer’s disease, cancers, and so on [50,51]. CRYAA is a substrate of UPP, and truncated mutant CRYAA was reported to undergo rapid degradation by UPP to prevent its accumulation in lens cells [29]. However, G98R CRYAA was only moderately degradable by UPP and prone to aggregation [19]. With TMAO treatment it appeared that TMAO either promoted the degradation of His/myc-G98R mutant through UPP or resolved the protein aggregation in the ER, hence reducing cellular stress. This was substantiated by reduced expression of the ER stress markers, PERK and BiP, as well as the pro-apoptotic proteins, caspase-3 and CHOP/GADD153. On the other hand, 3-MA treatment did not affect G98R CRYAA aggregation in cells suggesting that G98R CRYAA was not a substrate of autophagy.

TMAO is a naturally occurring osmolyte present in deep-water fishes. It is important to counteract the protein destabilizing effect of urea and hydrostatic pressure. Its action on protein folding or stabilization could be due to its hydrophobic nature to push water molecules to the protein surface. This increased hydration affects Gibbs free energy (G) levels along the protein-folding process so that ∆G between native and denatured states becomes larger and drives the equilibrium toward the native state [48]. The solvent-accessible surface area is reduced and causes a tighter packing of polypeptides or reduces the mobility between protein domains, leading to a more stabilized protein conformation and oligomeric assembly [52]. The mutated G98R CRYAA had exposed hydrophobic patches [19] which could be modified or removed by TMAO, resulting in a more stabilized conformation, likely recognized by UPP and were being degraded.

In addition, we uncovered another TMAO-initiated cytoprotection pathway that induced Hsp70 expression, which is critical for cell survival. Hsp70 is a molecular chaperone widely expressed in cells and functions to modulate the engagement and progression of apoptosis induced by various stimuli [53]. Hsp70 suppresses apoptosis by directly blocking the assembly of functional Apaf-1 apoptosomes [54]. It also helps proteins to fold through repeated cycles of substrate binding and release. The stress-inducible Hsp70 helps cells cope with adverse conditions, in part by aiding in the folding of nascent polypeptides or refolding damaged proteins, preventing or reversal of protein aggregation, or promoting protein trafficking [55-58]. We observed an improvement of mutant cell survival in our study. It appears that TMAO either stabilizes G98R CRYAA and promotes its UPP-associated degradation or modulates the protein-folding environment and reduces apoptosis through heat-shock response signaling. We could not determine if changes of Hsp70 expression were directly caused by TMAO treatment, which might involve a change of chaperoning capacity in cells, or a consequence of mutant protein stabilization. Our previous study also identified a delayed Hsp70 expression after heat-shock induction in R12C CRYAA-expressing cells [27]. Further work would provide better characterization of Hsp70 regulation in cataract pathogenesis and may introduce possible treatment strategies. The effect of TMAO on other cell stress signaling (including metal-induced stress and inflammatory stress) was not observed.

In conclusion, our study revealed cellular aggregation and ER stress induced by misfolded G98R mutant CRYAA in lens epithelial cells, and this caused apoptosis. These defects were alleviated by the natural osmolyte TMAO. Our findings provide a basis for a new chemical-based strategy to alleviate cell stress and improve cell survival.
